# Teaching Physiology-First Experience With Using a Simulator in Teaching Physiology of the Cardiovascular System

**DOI:** 10.33549/physiolres.935831

**Published:** 2026-08-01

**Authors:** Nikoleta MAZGUTOVA, Daniela MOKRA, Andrea CALKOVSKA, Jana PLEVKOVA, Zuzana BIRINGEROVA, Juraj MOKRY

**Affiliations:** 1 Department of Physiology, Jessenius Faculty of Medicine in Martin, Comenius University Bratislava, Martin, Slovakia; 2 Biomedical Center Martin, Jessenius Faculty of Medicine in Martin, Comenius University Bratislava, Martin, Slovakia; 3 Medical Education Support Center, Jessenius Faculty of Medicine in Martin, Comenius University Bratislava, Martin, Slovakiam; 4 Department of Anesthesiology and Intensive Medicine, Jessenius Faculty of Medicine in Martin and University Hospital Martin, Martin, Slovakia; 5 Department of Pharmacology, Jessenius Faculty of Medicine in Martin, Comenius University Bratislava, Martin, Slovakia

**Keywords:** CPS simulator, Cardiovascular system, Physiology

## Abstract

Simulation is widely used for training practical skills, clinical procedures, critical thinking, and communication. Its application in teaching cardiovascular physiology represents an innovative educational approach. We introduced the Cardiopulmonary Auscultation Patient Simulator (CPS) “Harvey” into the Physiology curriculum for second-year General Medicine students at Jessenius Faculty of Medicine, Comenius University. During practical sessions, students performed heart auscultation and blood pressure measurement tasks in two ways: classical investigation on each other or working with the CPS simulator in small groups (4–8 students). At the end of the practicals, students completed an anonymous questionnaire evaluating this teaching method. Respondents (n=110; 68 Slovak- and 42 English-program students) perceived the simulation-based teaching very positively. Most students reported that simulations improved their understanding of cardiovascular physiology topics previously covered in lectures. They found the activities easy to perform and expressed interest in broader use of simulation in other sessions. The main limitations reported were insufficient time for repeated practice and the relatively large group size. Students suggested longer sessions and smaller working groups. In conclusion, students considered the simulator-based practicals beneficial for learning and helpful for increasing confidence in practical skills. Based on their feedback, we plan to expand the use of simulations to additional physiology practicals and extend session duration to allow more hands-on practice.

## Introduction

Medical professions require a high level of mastery of procedural (technical) as well as non-technical (so-called soft) skills, which are a prerequisite for safe and professional provision of therapeutic and preventive care. For this reason, it is necessary to implement a method of practical teaching that allows this high level to be achieved by setting precisely defined goals in a sufficient scope, the necessary hourly allowance and the number of repetitions in a safe environment. The introduction of innovative and modern approaches to teaching the subject significantly contributes to increasing the level of internal motivation of students. Simulations represent such a method.

In recent years, simulations have become widely used in medical education to advance the acquisition of clinical skills, improve team working, and encourage deliberate practice in the clinical environment [1–3]. These methods represent complementary forms of education to often limited possibilities of clinical teaching providing a suitable link between acquisition of theoretical knowledge and training of clinical skills and clinical thinking, which is the basis for practising the profession of a doctor [4]. The simulations make the teaching process more attractive not only for students but also for teachers. Use of simulations enables the demonstration and explanation of topics in which the provision of direct practical experience is problematic, e.g. because of rare occurrence of certain diseases, operational reasons for clinics, nature of the subjects of study, *etc*. When using simulation techniques, a higher retention of knowledge has been repeatedly documented in comparison to classical teaching in the form of lectures, seminars and self-study [2]. Simulation is also helpful in the teaching process by strengthening the student’s interest in studying and improving in a particular field of medicine, such as Physiology [5].

In recent years, the use of simulation elements in the practical education of Physiology has gained significant traction, proving benefits in enhancing students’ understanding of complex physiological concepts.

Simulation-based learning provides an experiential environment that encourages active participation, allowing students to engage with physiological principles in a dynamic context. This active methodology fosters improved retention and understanding of intricate subject matter, which is often described as challenging in traditional education formats [6, 7]. Therefore, the active introduction of simulations into the curriculum appears to be a necessary step at all medical schools.

The aim of this pilot study was to assess the response of students of General Medicine studying in Slovak and English languages at Jessenius Faculty of Medicine, Comenius University (JFM CU) to introduction of simulation-based teaching methodologies in practical teaching of selected parts of cardiovascular physiology. Introduction of this „test simulation” aimed to detect any possible problem situations and provide the possibility to modify the scenario where needed. The other aim was to obtain the feedback from students on whether the simulations implemented into the curriculum improved student knowledge retention, clinical reasoning skills, and overall on boarding effectiveness to clinical practice in the context of cardiovascular system. This feedback from students will help to improve the simulation scenario for students in the next years.

## Methods

### Participants and research design

The present study was conducted in the week 8 (April 7 – April 11, 2025) of the education period in the summer term in pregraduate students of General Medicine studying in Slovak and English languages. The particular session described in this study took place in the Medical Education Support Center of JFM CU.

**Fig. 1:**
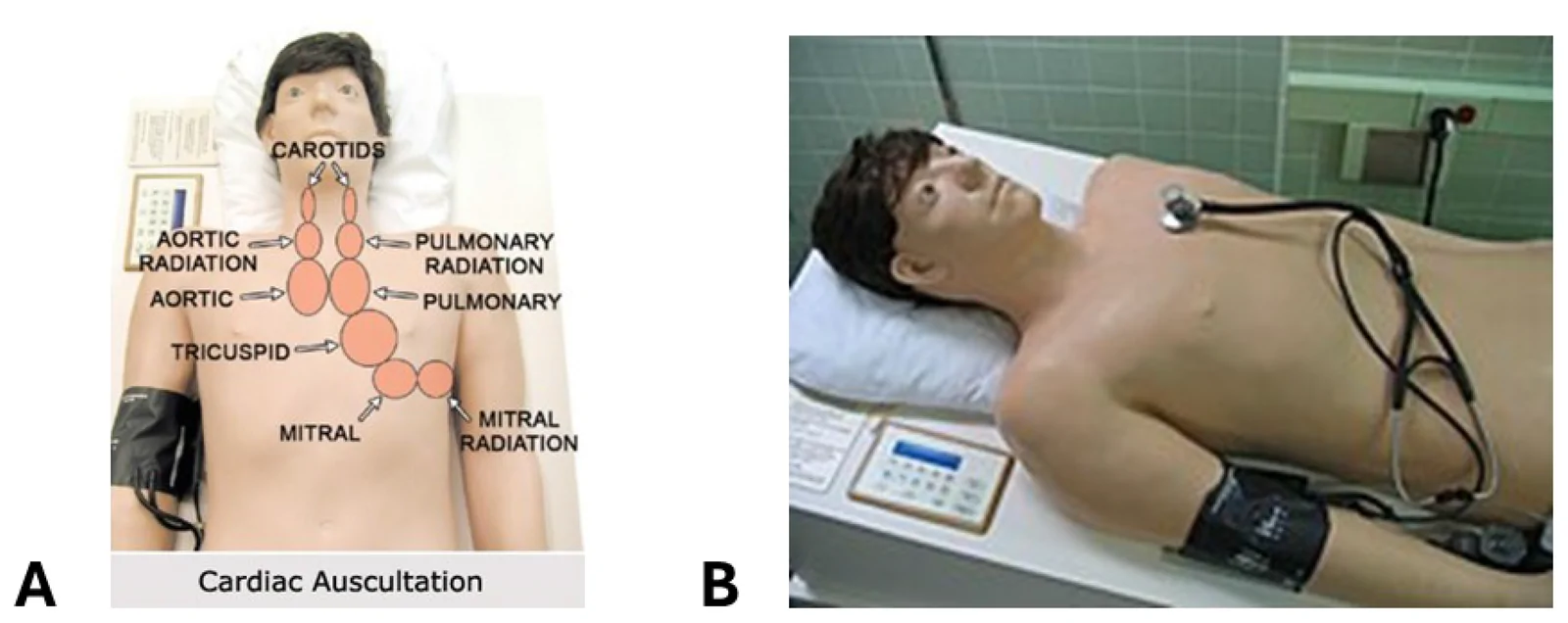
Cardiopulmonary Auscultation Patient Simulator “Harvey”. **A**) The training of heart auscultation on the mannequin at the standard places on the chest. **B**) The training of blood pressure measurement on patient simulator. Modified according to: [8, 9].

In this practical session, we used moderate fidelity CPS „Harvey” (Fig. 1). The CPS “Harvey” is a life-size patient simulator that allows realistic simulation of various cardiac findings and corresponding respiratory murmurs. The working with the CPS “Harvey” and its accompanying programs aims to help students acquire and improve skills in auscultation of the heart and lungs, better understand the symptoms and signs of cardiovascular diseases and thus learn to diagnose them correctly. After completing the training with the simulator, students will be able to better interpret the anamnesis of real patients, understand the physiology of the physical examination, in particular, improve their diagnostic skills in the physical examination of patients and will be able to formulate an appropriate initial diagnosis based on the initial examination of the patient [8].

### Course structure

At our faculty, medical students are taught cardiovascular physiology *via* lectures and practical sessions in the summer term of the 2^nd^ year of their study. The Physiology course consists of two lectures and a one practical session weekly, of which 6 consecutive weeks are dedicated to teaching the cardiovascular system. While lectures explain the function of the cardiovascular system and its interactions with other systems (e.g. respiratory system), practical exercises focus on gaining practical experience and skills in examining certain cardiovascular parameters. Students have the opportunity to investigate these parameters using various investigational methods commonly used in clinical practice or research. For most tasks, students work in pairs or small groups. The use of demonstrations of various investigational methods in the practical classes is intended to facilitate transfer of the theory learned during the face-to-face lecture into the practice. In addition, students requisite skill training on laboratory diagnostic methods. The simulation practical classes use both a „flipped classroom” learning (preclass self-study).

The original practical exercise included tasks for examining heart sounds by stethoscope and measuring blood pressure by auscultatory and oscillometric methods, which were measured by students on each other in pairs. In order to expand practical experience and skills, the content of this practical session was significantly modified. We have added several interactive tasks for students, including simulations of various clinical situations using the CPS Harvey simulator. In advance, students were provided with case notes and protocols, which were necessary for their self study before the laboratory class and answering the problem questions in the protocol. The proper practical exercise consisted of two tasks: „Auscultation of the heart sounds” (Task 1) and „Measurement of the blood pressure” (Task 2).

Task 1 included training of the heart auscultation at the standard places on the chest (i.e. auscultation of the mitral, tricuspid, aortic and pulmonary valve sounds) firstly on students (as a work in pairs) and then on the mannequin (Harvey simulator) (Fig. 1A). After familiarization with characteristics of basic heart sounds and murmurs on the mannequin, students underwent training of following physiological and pathological auscultation findings: “physiological 2 sounds”, “physiological 3 sounds”, “systolic murmur”, “diastolic murmur” or “continuous murmur”, which were selected in a random order from the menu of Harvey simulator.

Task 2 included measurement of the blood pressure in students (as a work in pairs). Students trained measurement of the blood pressure using the auscultation and oscillometric methods and compared the results measured by these methods on the right and left uppear limbs. Then, students compared the values of the blood pressure measured with an electronic blood pressure monitor in the following situations resembling common situations in the clinical practice: 1. blood pressure was measured after the examined person was sitting still, without moving, without talking for 3 min, 2. blood pressure was measured immediately after the examined person walked at least 20 steps around the room and sat on a chair, 3. blood pressure was measured during an active conversation of the examined person with the examiner (simulation of an active conversation with a doctor). For training the blood pressure measurement in the pathological conditions, three different situations were selected in a random order from the menu of Harvey simulator (Fig. 1B): a) normotension, b) hypotension, c) hypertension.

The students were divided into 2 subgroups containing 4–8 persons in Room 1 without the simulator and in Room 2 with the CPS Harvey simulator. Thus, students in one subgroup trained physical assessment skills of measurement of blood pressure and auscultation of the heart sounds on each other (a work in pairs), while students in the other subgroup trained the measurement of blood pressure and auscultation of the heart sounds on the patient simulator. After finishing their tasks, the student groups alternated. The duration of the adjusted practical session based on the simulations was 90 min in total.

**Fig. 2:**
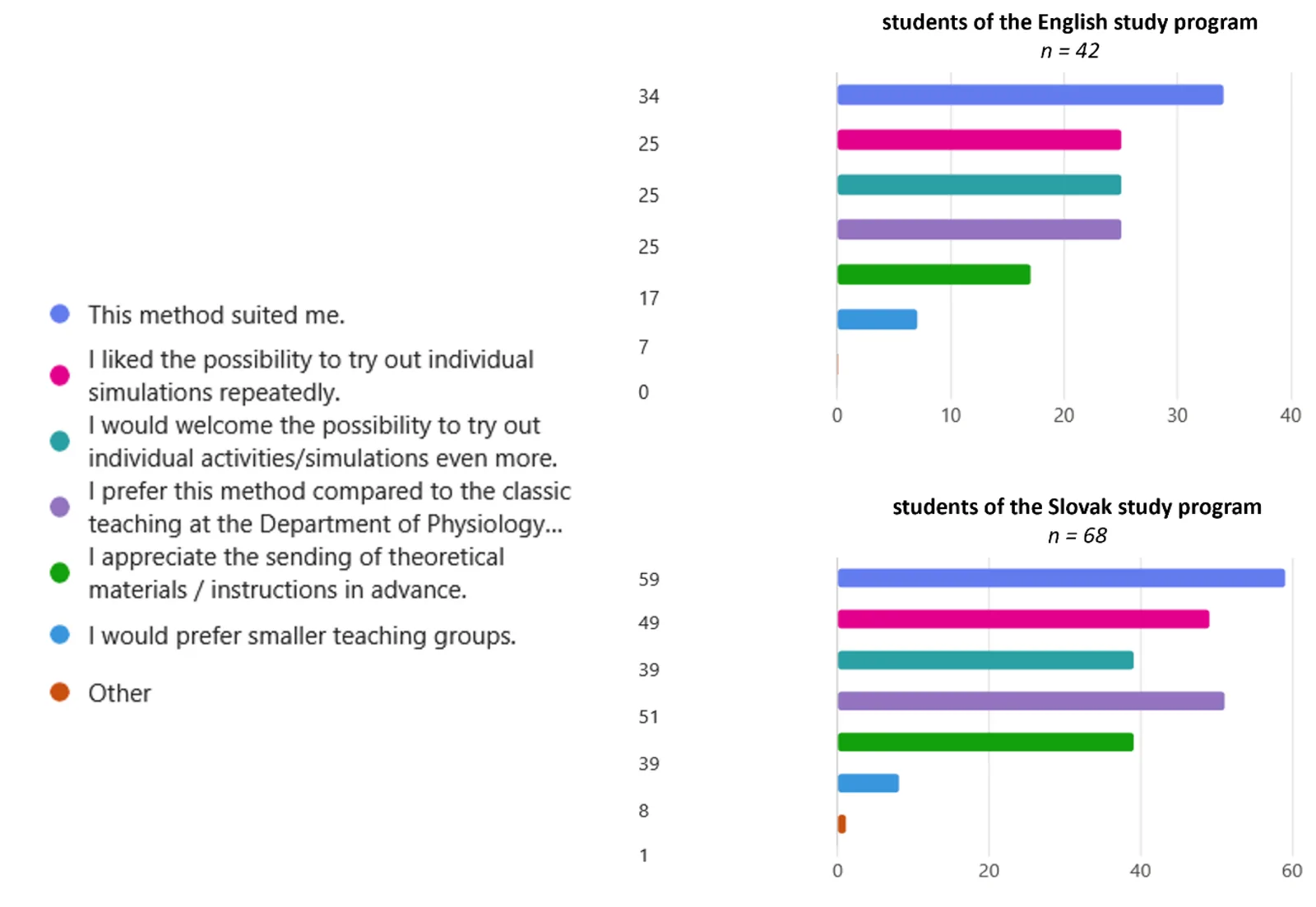
Summary of evaluation of the modified practicals by medical students of the Slovak and English study programmes. **Question A)** Please indicate your level of satisfaction with individual areas related to teaching practical exercises in physiology in the simulation center. **Question B)** How did you perceive the teaching in the simulation center? (*Students could select more than one option*).

At the end of the practical session, students were asked to participate in an anonymous online questionnaire about their experience with this renewed approach to learning. The questions in the questionnaire asked whether students thought the simulation experience had benefited their learning. Open-ended questions were also included. The questions clusters and related aspects of the student experience tested in this evaluation are shown in Figure 2A, Figure 2B and in the text below.

### Analyses

The open-ended questions were analyzed using a qualitative content analysis to identify themes associated with the most and least effective aspects of the simulation-based learning experience. Authors of this pilot study met to discuss their findings to gain consensus on themes.

## Results

### Student feedback on the learning experience

The results of the evaluation are shown in Figures 2A and 2B. Response rates were 54.4 % (n=68 responding students from 125 students in total) in students in the Slovak language program and 31.1 % (n=42 responding students from 135 students in total) in students in the English language program.

Responses to open-ended questions were mostly positive and constructive. Of the students who provided comments, responses ranged from a few words to complete sentences. The nature of these comments non-differed between student’s cohorts; however, words and phrases including “helpful,” “enjoyable,” “the perfect form of education,” “more frequent using of the simulation in physiology education” and „good experience” were common. Another common theme among student comments related to a sense that the simulations enhanced their learning:

*„It demonstrated how theoretical knowledge is applied in a hospital setting, which was simulated.”*

*„I’d rather have more simulation classes than theoretical classes.”*

*„It makes the process of learning more interesting and engaging.”*

*„It was really good to be able to apply knowledge instead of just remembering the facts. I can remember the things like blood pressure and others better now. It was really beneficial to apply the knowledge I had gained in lectures to the practical experience.”*

*„It would certainly be appropriate to repeat similar practicals associated with the simulations more frequently (for example, three times per semester after conclusion of the theoretical unit).”*

*„I liked that we could practisize on the good model as well as on each other”*.

*„The practising on the manikin as well as the other tasks were perfect.”*

*„I liked using the manikin and learning environment in the simulation center*.

*„I appreciate that teachers sent the study material in advance. It made me prepared. Demonstrators were really helpful.”*

As the results showed, students perceived this method of education very positively. Among the student cohorts, there was overwhelming support for the simulations as a means of achieving better understanding of the selected chapters from cardiovascular physiology taught in the lectures. Students felt that the simulations were easy to undertake, and they welcomed a greater use of technology in the course. They also strongly expressed that the simulation provided them with an experience similar to working as a health professional, increasing their confidence in practical skills in the same time.

However, students considered duration of the practicals for not sufficiently long to practicize the tasks on heart auscultation and blood pressure measurement in pairs (Room 1) or on the patient simulator (Room 2) repeatedly. Moreover, students would welcome the possibility to work in even smaller groups. These results are also confirmed by the verbal comments, in which the students of both programmes comprehensively agreed:

*“We would appreciate a longer duration of practice in this form.”*

*“I would prefer smaller teaching groups.”*

## Discussion

In this pilot study, we evaluated the students’ response to introducing simulations into selected parts of the cardiovascular system in Physiology course in the second year of studying General Medicine. The results support the notion that students’ engagement and interest increase when simulations are integrated within the curriculum. This active learning approach is supported by findings demonstrating how the practical exercises designed thoughtfully can lead to improved comprehension of complex physiological functions [6]. The positive primary results of this study can also be partially explained by high levels of motivation. Benefits for future practice include learning to work in teams, awareness of effective communication strategies, and appreciating the holism of practice [10, 11].

Students have prepared for the meeting through lectures and online materials, which they had available before this practical exercise in the JFM CU. Students in the questionnaire appreciated the sending of theoretical materials/instructions in advance. Students then practiced the tasks - heart auscultation and blood pressure measurement - on a patient simulator in small groups or on themselves in pairs. Through practicing physiological assessment (auscultation of the heart sounds and blood pressure) on the mannequin or on each other, students assumed health professional roles.

In the feedback after the simulation educational activity, the vast majority of students stated that they would welcome the possibility to try out individual activities/simulations even more. They appreciated the possibility of trying out individual simulations repeatedly. The possibility of repeated practice of a simulated performance as many times as the student needs is one of the most significant advantages of the simulation method. Every student has individual prerequisites and needs for mastering a particular performance/skill, but every student needs different time and different number of repetitions to master it flawlessly [12]. This finding is supported by several studies [13, 14, 15] that clearly demonstrate the positive impact of repeated training in a simulated environment on the quality of students’ performances.

Adoption of more simulation activities within health care education has been influenced by advances in the technological capabilities of mannequins and driven by patient safety agendas [10, 16, 17, 18]. The simulations in medical education have been shown to significantly enhance learning outcomes compared to traditional educational methods. A meta-analytic review by McGaghie et al. indicates that simulation-based medical education combined with deliberate practice supports superior educational outcomes, particularly in clinical settings where hands-on skills are critical for professional competence [2]. Specifically, high-fidelity simulation allows students to engage with realistic clinical scenarios that enhance their learning experience, providing opportunities to practice procedural and decision-making skills in a safe environment [3, 4, 19]. At a minimum, the contextual nature of a scenario experienced within lifelike settings allows participants to act out what they know and learn from errors without consequences to the “patient,” in essence enabling experiential learning within a community of practice [10].

The concept of “self-simulation” has also emerged as a relevant teaching method, where students actively simulate physiological processes themselves, further empowering them to engage with the material [20]. A study by Zheng et al. emphasizes the positive impact of the simulation on learning experience and outcomes in a cardiovascular physiology context, signifying that the structured integration of simulation into teaching methodologies can significantly enhance knowledge retention and application [21]. Similarly, the study of Helyer and Dickens elaborates on the successful incorporation of various simulation scenarios alongside traditional teaching methods in physiology and pharmacology education, suggesting that the fusion of different pedagogical approaches optimizes the learning environment [22]. In a cohort study on medical students from five institutions, one group was exposed to 2 weeks of deliberate practice of cardiac bedside skills using the CPS “Harvey” followed by 2 weeks of traditional ward work, while the other group went through 4 weeks of traditional ward training. The simulation group performed at twice as well as the ward group, with only half the training time [23]. DeVita et al. showed better outcomes in simulated patients if doctors were trained to work together by reliably performing preassigned roles during a simulator exercise [24]. Bintley et al. asserted that although simulation tools are underutilized, they possess the potential to reinforce key physiological concepts and enhance clinical reasoning skills in medical education [7]. These methods, when integrated into the cardiovascular physiology curriculum, can effectively bridge the gap between theoretical knowledge and practical application, giving the complexity of cardiovascular medicine [21, 25].

Aside from enhancing content understanding, simulations in Physiology courses also correlate with increased confidence in clinical practice. For example, Mannella et al. found that simulation of childbirth improved medical students’ clinical management capabilities and self-confidence, reflecting the broader applicability of simulation in nurturing healthcare competencies across various medical disciplines [26]. However, it is still worth noting that a gap exists between the reality and simulation laboratory experience regardless of how authentic it is.

## Conclusion

In conclusion, our findings clearly indicate a positive feedback loop for the implementation of simulations in the training of students of General Medicine. Simulation plays an important factor in the evaluation of education environment, which optimizes teaching by reflecting both positive and negative aspects of education and related influences.

The integration of simulation elements into Physiology course not only augments traditional teaching methodologies but also enhances student engagement, understanding, and competence in practical applications. The accumulation of evidence underscores the imperative need for developing pedagogical frameworks that embrace simulation as a foundational element of Physiology education, thereby preparing students effectively for the challenges of clinical practice.

## Limitations

There are several limitations of this pilot study that need to be explored further or to be solved. First, several students complained that they did not have enough time to repeat tasks multiple times, especially on the simulator. In this pilot study, duration of the practical session was limited to 90 minutes in total because of space and time limitations in the simulation center. However, taking the students’ comments into account for the next year we plan to extend the duration of the practical to 120 minutes. Students will have significantly more time for performing individual simulations on the simulator and each other in pairs, thus consolidating the knowledge they acquired in the theoretical part of the Physiology. Secondly, the present data indicate the acquisition of short-term gains, but do not provide information on longer-term knowledge retention. Third, the future goal is to determine whether this approach is useful to develop the integration of basic and clinical science and as a learning tool for the students to bridge the gap between theoretical knowledge and practical skills. Our study did not directly address the effectiveness of the simulation on improving the student’s ability to apply their knowledge in practice. Fourth, the survey response rate was 54.4 % in students in the Slovak language program and 31.1 % in students in the English language program, which despite being an adequate sample size to capture the overall range of feedback, can still run the risk of biased sampling. Last, this study was conducted on a relatively small number of student participants from one study year from one institution, thus limiting the generalization of the findings.

## Educational Implications

This study was our first endeavor of integration of simulations into Physiology curriculum to enhance clinical reasoning, clinical exposure, and cardiovascular concept mastery. High student motivation and core features of quality simulation-based learning activities contributed to student perceptions of the value of this activity. There may be similar opportunities in other aspects of the curriculum where we can leverage more clinical technologies to further enhance learning. Expanding to the other complex organ systems can generate further empirical evidence to better inform future simulation-enhanced educational endeavors. Students’ high level of enthusiasm and acceptance validated its meaningful incorporation into educational curricula to build a solid foundation for their future success as doctors/physicians.
